# Immune-regulating extracellular vesicles: a new frontier in autoimmune disease therapy

**DOI:** 10.1042/EBC20253016

**Published:** 2025-05-13

**Authors:** Hassan Shah, Zhengkun Liu, Weisheng Guo, Wenjie Ren, Yafang Xiao

**Affiliations:** 1 Department of Minimally Invasive Interventional Radiology, The Second Affiliated Hospital, School of Biomedical Engineering, Guangzhou Medical University, Guangzhou 510260, China; 2 Institutes of Health Central Plain, Clinical Medical Center of Tissue Engineering and Regeneration, Xinxiang Medical University, Xinxiang 453003, China; 3 Department of Cardiology, Guangzhou Institute of Cardiovascular Disease, Guangdong Key Laboratory of Vascular Diseases, Guangzhou Medical University, Guangzhou 510260, China

**Keywords:** autoimmune disease, cell-based therapy, extracellular vesicles, immune regulation

## Abstract

Immune regulation is recognized as a cornerstone therapeutic strategy for the treatment of various autoimmune diseases. These disorders, driven by dysregulated immune responses, contribute significantly to morbidity and mortality. Although conventional immunosuppressive therapies provide symptomatic relief, their prolonged use is often associated with severe adverse effects, underscoring the need for safer and more effective treatment approaches. Extracellular vesicles (EVs), derived from immunoregulatory cells such as regulatory T cells, dendritic cells, mesenchymal stem cells, and neutrophils, have emerged as promising candidates for targeted immunomodulation. These nanoscale vesicles inherit the immunosuppressive properties of their parental cells, thereby facilitating immune homeostasis while mitigating the risks associated with other cell-based therapies. This review provides a comprehensive overview of recent advances in the application of immunoregulatory cell–derived EVs for autoimmune disease treatment, with a particular focus on their mechanisms of action within the immune microenvironment. Finally, we discuss the challenges and potential future directions in the development of EV-based therapies for autoimmune diseases.

## Introduction

Autoimmune diseases, characterized by dysregulated immune responses, constitute a major contributor to morbidity and mortality among chronic illnesses. Aberrant immune activation results in the production of autoantibodies, leading to progressive tissue damage [1,2]. Although immunosuppressive therapies provide temporary symptomatic relief, their prolonged administration is associated with an increased risk of opportunistic infections and corticosteroid-induced metabolic dysfunction, thereby elevating the likelihood of cardiovascular complications [3,4]. In response to these limitations, cell-based therapies have gained significant attention due to their potential to modulate immune responses and restore immune homeostasis. Moreover, advancements in elucidating the molecular mechanisms and signaling pathways underlying autoimmune disease pathogenesis have further facilitated the application of heterogeneous cell populations to correct immune system imbalances [5].

**Figure 1:**
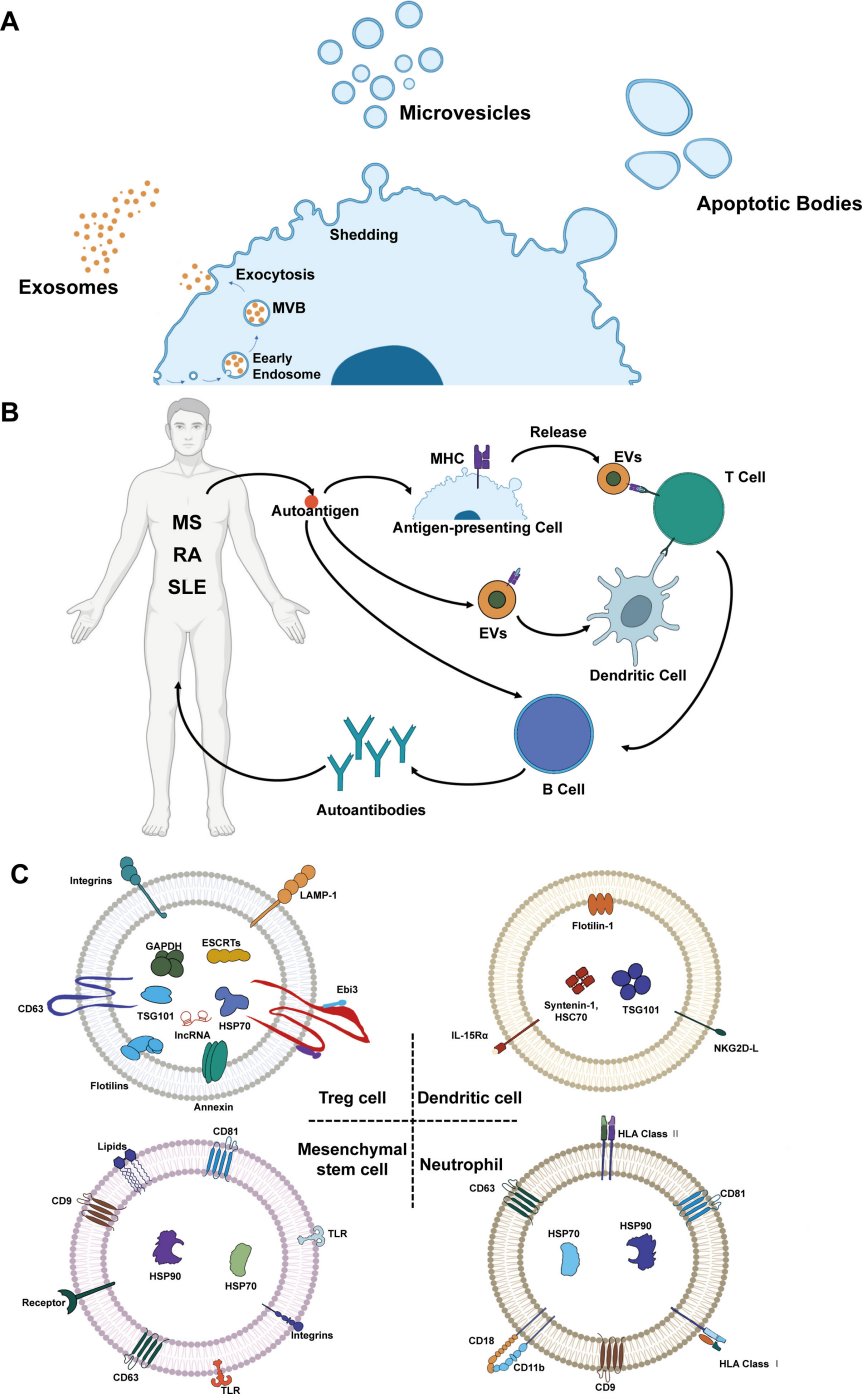
Extracellular vesicle (EVs) in autoimmune diseases. (**A**) Classification of EVs into various types on the basis of biogenesis, including exosomes, microvesicles, and apoptotic bodies. (**B**) EVs in immune modulation of autoimmune diseases like multiple sclerosis, rheumatoid arthritis, and systemic lupus erythematosus. (**C**) Various types of EVs, such as Treg-derived EVs, dendritic cell-derived EVs, mesenchymal stem cell-derived EVs, and neutrophil cell-derived EVs showing their major components.

Extracellular vesicles (EVs) have emerged as potent immunomodulators and promising alternatives to conventional cell-based therapies due to their distinct molecular signatures and diverse physiological functions [6–9]. Based on their biogenesis and size, EVs are broadly classified into exosomes (30–150 nm), microvesicles (100–1,000 nm), and apoptotic bodies (1,000–5,000 nm) (Figure 1A) [10,11]. Secreted by various cell types, EVs play a crucial role in intercellular communication by interacting with target cell receptors and initiating signaling cascades that modulate cellular behavior and physiological processes. EVs are involved in many autoimmune diseases, such as multiple sclerosis (MS), rheumatoid arthritis (RA), and systemic lupus erythematosus (SLE), characterized by loss-tolerance and autoantibody production targeting self-tissues. The autoantigen released from damaged tissues can recognized by immune cells triggering a cascade of the event and initiation of autoimmune disease (Figure 1B) [12–14]. Notably, EVs derived from immunoregulatory cells inherit the immunomodulatory properties of their parental cells. For example, regulatory T cell (Treg)-derived EVs are enriched with transforming growth factor-beta (TGF-β) and interleukin-10 (IL-10), conferring a distinct advantage in suppressing autoimmune responses. Moreover, their intrinsic tissue-homing capabilities enable precise targeted delivery while mitigating the tumorigenic risks associated with cell-based therapies [8,15]. Therefore, EV-based therapies hold significant potential for the treatment of autoimmune diseases, providing novel avenues for targeted immunomodulation [16].

Herein, we review the latest advances in the use of immunoregulatory cell–derived EVs for the treatment of autoimmune diseases. Specifically Figure 1C depicts the EVs derived from Tregs, dendritic cells (DCs), neutrophils, and mesenchymal stem cells (MSCs), as well as their mechanisms of interaction with the immune microenvironment in autoimmune diseases. The various immune regulating cell derived EVs contains fusion proteins and membrane transporters (annexin and flotilins), cell adhesion proteins (integrins), lysosomal related membrane proteins, heat shock proteins (HSP70 and HSP90), endosomal sorting complex for transport, auxiliary protein components (TSG101), membrane proteins (integrins, CD9, CD18, CD11b, CD 63), nucleic acids (IncRNA), lipids, and human leukocyte antigen. Furthermore, the molecular and cellular pathways through which these EVs exert their therapeutic effects are discussed, highlighting their role in modulating antigen presentation, cytokine secretion, and immune cell differentiation. Finally, we critically analyze the challenges associated with the clinical translation of immunoregulatory cell–derived EV therapies, including scalability, standardization, and potential off-target effects. We also explore future directions for optimizing EV-based therapeutic strategies, such as engineering approaches to enhance targeting specificity and bioactivity.

## Immune-regulating cell derived EV-based therapy in autoimmune diseases

EVs derived from immunoregulatory cells have emerged as a promising therapeutic platform for autoimmune diseases due to their capacity to modulate immune responses, restore immune tolerance, and serve as carriers for therapeutic cargo. Their ability to influence key immunological processes, including antigen presentation, cytokine secretion, and immune cell differentiation, underscores their potential as targeted immunomodulatory agents. Furthermore, advancements in elucidating the molecular mechanisms and signaling pathways underlying the pathogenesis and progression of autoimmune diseases have facilitated the development of EV-based strategies aimed at re-establishing immune homeostasis and correcting dysregulated immune responses [5]. Notably, EVs derived from immunoregulatory cells, such as Tregs, DCs, neutrophils, and MSCs, have demonstrated significant potential in autoimmune disease treatment by exerting multifaceted immunosuppressive effects, while minimizing the risks associated with traditional cell-based therapies. Consequently, EV-based therapies represent a promising avenue for precision immunotherapy, offering targeted and potentially safer alternatives for managing autoimmune disorders.

### Treg-derived EVs

Tregs constitute a specialized subset of T lymphocytes that play a pivotal role in maintaining immune tolerance and homeostasis [17]. By actively suppressing excessive immune responses, Tregs mitigate aberrant autoreactivity, thereby preventing the onset and progression of autoimmune diseases while preserving self-tolerance. Notably, EVs derived from Tregs (Treg-EVs) have been identified as a sophisticated, cell-contact-independent mechanism through which Tregs exert their immunosuppressive effects. These nanoscale vesicles encapsulate a diverse repertoire of bioactive molecules, including immunoregulatory cytokines, microRNAs, and surface ligands, which collectively modulate the activity of recipient immune cells. Moreover, the intrinsic ability of Treg-EVs to facilitate targeted delivery to specific immune microenvironments enhances their therapeutic potential in the management of autoimmune diseases. Given their capacity to mimic key immunosuppressive functions of their parental cells while circumventing challenges associated with direct Treg cell therapy, Treg-EVs have emerged as a promising avenue for the development of precision immunotherapies [18].

The interaction between regulatory Treg-EVs and target cells is mediated through multiple mechanisms, including signal transduction and molecular transfer via binding, membrane fusion, and endocytosis. Upon receptor-mediated fusion with the target cell membrane, Treg-EVs release their bioactive cargo into the cytoplasm, facilitating the transfer of transmembrane proteins, lipid components, and regulatory molecules [19,20]. Moreover, Treg-EVs exert potent immunomodulatory effects by suppressing T-cell proliferation, modulating cytokine secretion, and inducing apoptosis in effector immune cells, thereby contributing to immune tolerance and homeostasis [21–25].

Regulatory Treg-EVs have been shown to carry key immunosuppressive molecules, including cytotoxic T-lymphocyte-associated protein 4 (CTLA-4) and TGF-β, which play pivotal roles in immune regulation. In addition, immunomodulatory markers such as CD25 and CD73 have been identified within Treg-EVs, with CD73 recognized as a critical mediator of Treg-associated immunosuppressive functions [26,27]. Azimi et al. [28] demonstrated the immunomodulatory effects of Treg-derived exosomes in the treatment of MS, a condition in which an imbalance in Treg function is implicated in disease pathogenesis. In their study, CD63^+^ exosomes were isolated from Tregs of both control and healthy groups and subsequently co-cultured with conventional T cells (CD4^+^CD25⁻). The results indicated that Treg-derived exosomes suppressed T-cell proliferation in patients with MS, albeit with reduced apoptotic induction compared with the control group. Additionally, multiple studies have reported defective immunomodulatory functions of CD4^+^CD25⁻ Tregs in patients with MS, further underscoring the role of Treg dysregulation in disease progression [29]. Similarly, Chen et al. [30] investigated the therapeutic potential of TGF-β-induced CD4^+^FoxP3^+^ Treg-EVs in a murine model of RA. Their findings demonstrated that Treg-EVs preferentially targeted inflamed joints and effectively mitigated Treg imbalances in arthritic mice. However, despite their immunosuppressive potential, Treg-EVs were observed to suppress FoxP3^+^ Treg cell functions, which may influence overall immune homeostasis. Moreover, the inflammatory microenvironment within affected joints—characterized by elevated levels of pro-inflammatory cytokines—could attenuate the immunosuppressive efficacy of Treg-EVs, thereby allowing pathogenic effector T cells to sustain inflammation. Notably, an abundance of FoxP3^+^ T cells has been detected in inflamed joints, where they appear to contribute to the persistence and exacerbation of inflammatory responses, highlighting the complex interplay between Treg-EVs and the inflammatory milieu in autoimmune disease progression.

### DC-derived EVs

DCs serve as central regulators of immune responses and have emerged as a promising target for immunomodulation in the treatment of autoimmune diseases. As highly specialized antigen-presenting cells, DCs play a crucial role in bridging innate and adaptive immunity by processing and presenting antigens to naïve T cells through major histocompatibility complex molecules. This antigen presentation process is fundamental to T-cell activation and the subsequent initiation of downstream immune responses. Notably, DCs constitute a heterogeneous population characterized by distinct phenotypic markers, anatomical localization, and functional specialization within the immune system [31,32].

EVs derived fromDC-EVs are enriched with immunomodulatory molecules that selectively target specific immune cell subsets, presenting significant therapeutic potential for the management of autoimmune diseases. Within the context of autoimmunity, DCs exhibit dual functionality: while they are capable of initiating adaptive self-reactive immune responses, they also play a pivotal role in promoting and maintaining immune tolerance. DCs secrete various anti-inflammatory mediators, including CTLA-4, TGF-β, IL-10, interleukin-4, and Fas ligand, all of which contribute to immune regulation. The immunomodulatory functions of DCs are mediated through key mechanisms, such as antigen presentation, cytokine secretion, maturation, and activation, facilitating interactions with various immune cell types, including T lymphocytes, B lymphocytes, and Tregs [33,34]. Manni et al. [35] investigated the interaction between DCs and EVs derived from amniotic fluid stem cell lines, providing critical insights into the selective uptake and functional consequences of DC-EVs. Their study demonstrated that DC-EVs were preferentially internalized by specific DCs subsets, particularly conventional DC2 cells, through CD29 receptor-mediated endocytosis. This selective uptake led to a marked reduction in the expression and secretion of pro-inflammatory mediators by tolerogenic DC subtypes in a murine model of multiple sclerosis. These findings underscore the therapeutic potential of DC-EVs in restoring immune homeostasis and highlight promising avenues for the development of EV-based therapies for autoimmune diseases.

### Neutrophil cell-derived EVs

In the rapidly evolving field of EV research, neutrophil-derived EVs (N-EVs) have garnered significant attention due to their distinctive immunomodulatory properties, encompassing both anti-inflammatory and pro-resolving functions. They exert their anti-inflammatory effects by suppression of T-cell activation, and scavenging pro-inflammatory molecules, whereas, pro-inflammatory role by promoting cytokine production, endothelial cell activation, enhancement of immune cell recruitment, NETosis, and pathogen clearance. As the most abundant leukocytes in the human body, neutrophils play a fundamental role in innate immunity, serving as the first line of defense against pathogenic infections. Notably, N-EVs actively contribute to immune regulation by exerting both immunosuppressive and immunostimulatory effects, thereby influencing the dynamic balance between inflammation and resolution. These nanoscale vesicles are considerably smaller than their parental neutrophils, lack the capacity for self-replication, and do not contain functional nuclei. Their biogenesis occurs through two primary mechanisms: direct shedding from the plasma membrane and release via independent intracellular pathways [36–38].

Neutrophils execute their immunological functions through multiple mechanisms, including degranulation, phagocytosis, and the formation of neutrophil extracellular traps (NETs) in response to pathogen recognition [39,40]. N-EVs are enriched with bioactive molecules such as chemokines, cytokines, and lipid mediators, all of which play crucial roles in the pathophysiology of various autoimmune diseases. Notably, NET formation has been identified as a key factor in both the initiation and exacerbation of systemic autoimmune diseases. By exposing autoantigens and promoting immune activation, NETs contribute to sustained inflammatory responses, leading to tissue and organ damage. The excessive generation of NETs can enhance autoantigen availability, thereby triggering aberrant immune responses in genetically or environmentally susceptible individuals [5,41]. Rhys et al. [42] investigated the immunoregulatory properties of N-EVs in patients with RA, specifically assessing their potential to modulate macrophage activation and inflammatory responses. The study compared N-EVs derived from neutrophils of healthy individuals with those from patients with RA, analyzing their effects on human monocyte-derived macrophages and identifying molecular factors involved in macrophage polarization. Notably, upon stimulation with tumor necrosis factor, neutrophils rapidly released microvesicles, a subset of which expressed pro-resolving proteins, including annexin-1 and phosphatidylserine. These proteins actively counteracted macrophage activation and facilitated the secretion of TGF-β, thereby contributing to the resolution of inflammation. These findings underscore the potential of N-EVs as a novel therapeutic approach for regulating excessive immune activation in autoimmune diseases.

### MSC-derived EVs

MSCs are multipotent progenitor cells residing in various tissues, characterized by their potent immunosuppressive properties and immune-privileged status. Through intricate interactions with the immune system, MSCs play a crucial role in maintaining immune homeostasis and reinstating immune tolerance, thereby mitigating aberrant immune activation. These immunomodulatory capabilities position MSCs as a promising therapeutic candidate for the treatment of immune-related disorders, including autoimmune diseases. Further investigation into the molecular mechanisms underlying MSC-mediated immunoregulation will be essential for optimizing their clinical application in immune-modulating therapies [43].

EVs derived from MSC-EVs contain a diverse array of bioactive molecules, including lipids, proteins, and nucleic acids, which play a critical role in intercellular communication and immune response modulation. MSC-EVs are increasingly recognized as an effective, cell-free therapeutic modality for the treatment of autoimmune diseases, offering significant advantages over direct MSC transplantation in terms of safety and scalability [44]. These vesicles exert immunomodulatory effects on a wide range of immune cells, including T and B lymphocytes, DCs, natural killer cells, and macrophages. Additionally, MSC-EVs influence the regulation of cytokines and chemokines, thereby facilitating the recruitment and activation of immune cells at sites of tissue injury or inflammation. MSC-EVs have demonstrated substantial anti-inflammatory properties in autoimmune diseases, such as RA, where they contribute to the suppression of inflammatory responses and promote the regeneration of anti-inflammatory cytokines, particularly IL-10 [45,46]. By enhancing IL-10 production and reducing the levels of pro-inflammatory cytokines, such as tumor necrosis factor-alpha, MSC-EVs help to restore balance in the immune system [11,47]. In an innovative approach, Xu et al. [48] engineered PD-L1-overexpressing MSC-EVs using lentivirus-mediated gene transfection, aiming to reconfigure the immune microenvironment in tissues affected by autoimmune diseases. These PD-L1-enriched MSC-EVs, as a cell-free therapeutic platform, effectively targeted and restored tissue integrity by inhibiting inflammatory immune responses via the PD-L1 pathway. The targeting of immune checkpoints, such as PD-L1, has increasingly gained recognition as a promising therapeutic strategy not only in cancer but also in autoimmune and inflammatory disorders.

In a study conducted by Tieu et al. [49], *in vivo* preclinical investigations were performed to assess the therapeutic potential of MSC-EVs in animal models. Similarly, Chen et al. [50] explored the therapeutic efficacy of MSC-derived miRNA-exosomes for the treatment of RA. Specifically, the miRNA-150-5p-expressing MSC-derived exosomes were found to influence the expression of matrix metalloproteinase-14, vascular endothelial growth factor (VEGF), as well as fibroblast-like synoviocytes (FLS) migration, invasion, and angiogenesis *in vitro*. Furthermore, a collagen-induced arthritis model was established to evaluate the *in vivo* effects of these exosomes. MSC-150 exosomes reduced FLS migration and invasion in the RA model and down-regulated tube formation in human umbilical vein endothelial cells by targeting MMP14 and VEGF. Additionally, the exosomes mitigated joint destruction by inhibiting synoviocytes hyperplasia and angiogenesis. These findings demonstrate that MSC-derived exosomes facilitate the intracellular transfer of miRNAs between cells, suggesting a promising therapeutic strategy for RA treatment. Overall, the study highlights the considerable potential of MSC-EVs for treating a wide range of diseases with minimal side effects, underscoring their promise as a novel therapeutic approach.

## Outlook

EVs play a pivotal role in maintaining the stability of the immune system and hold considerable promise for the treatment of autoimmune diseases. They represent an innovative and transformative approach to modulating immune responses and restoring immune homeostasis. The engineering approaches includes pre-isolation, post-isolation, and its subsequent modification of these EVs. Pre-isolation techniques include genetic, metabolic, and parent-cell membrane engineering, whereas, post-isolation techniques include physical and chemical engineering modification. Notably, EVs can be customized to meet the specific needs of individual patients, thereby facilitating personalized therapeutic interventions that optimize therapeutic efficacy while minimizing systemic toxicities. Furthermore, EVs can be administered through non-invasive routes, such as oral, inhalation, or topical delivery, potentially enhancing patient compliance with treatment protocols.

However, several challenges remain in the clinical translation of EV-based therapies. First, the efficacy of EV-based therapies is still hindered by insufficient targeting efficiency and the potential leakage of therapeutic payloads [51–53]. Second, standardization poses a significant concern. The main concern regarding standardization process includes batch-to-batch variability and standardization requirements. Batch-to-batch variability includes variation in isolation methods and source cell, its storage and handling techniques and inconsistency in its quantification methods, and regulatory consideration includes the lack of regulatory standard guidelines to encourage the best practices and good manufacturing practices. The isolation, purification, and characterization of EVs require the development of rigorous and standardized protocols to ensure reproducibility and therapeutic consistency. Inadequate standardization could lead to variability in EV preparation, resulting in inconsistent therapeutic outcomes in the treatment of autoimmune diseases. Finally, although EVs are generally regarded as having low immunogenicity, a comprehensive evaluation of their long-term safety, particularly in chronic therapeutic applications, remains essential. Addressing these challenges will be crucial for fully realizing the therapeutic potential of EVs in immune modulation and the management of autoimmune diseases.

**Summary**

- EVs are membrane-bound vesicles released by various cell types, recognized as sophisticated immunomodulators and promising alternatives to traditional cell therapies.
- Immune-regulating EVs show significant potential in treating autoimmune diseases, such as multiple sclerosis, SLE, and RA.
- We reviewed EVs derived from Tregs, DCs, neutrophils, and MSCs, focusing on their mechanisms of interaction with the immune microenvironment in autoimmune diseases.
- We highlighted the crucial role of EVs in maintaining immune system stability and explored their potential for treating autoimmune diseases, as well as the key challenges in the clinical translation of EV-based therapies.

## Abbreviations

CTLA-4: Cytotoxic T-lymphocyte-associated protein 4
DCs: Dendritic cells
EVs: Extracellular vesicles
FLS: Fibroblast-like synoviocytes
IL-10: Interleukin-10
MS: Multiple sclerosis
MSCs: Mesenchymal stem cells
NETs: Neutrophil extracellular traps
RA: Rheumatoid arthritis
SLE: Systemic lupus erythematosus
TGF-β: Transforming growth factor beta
VEGF: Vascular endothelial growth factor
